# *Diaporthe* species causing stem gray blight of red-fleshed dragon fruit (*Hylocereus polyrhizus*) in Malaysia

**DOI:** 10.1038/s41598-021-83551-z

**Published:** 2021-02-16

**Authors:** Abd Rahim Huda-Shakirah, Yee Jia Kee, Kak Leong Wong, Latiffah Zakaria, Masratul Hawa Mohd

**Affiliations:** School of Biological Sciences, Universiti Sains Malaysia, 11800 Penang, Malaysia

**Keywords:** Fungi, Sequencing

## Abstract

This study aimed to characterize the new fungal disease on the stem of red-fleshed dragon fruit (*Hylocereus polyrhizus*) in Malaysia, which is known as gray blight through morphological, molecular and pathogenicity analyses. Nine fungal isolates were isolated from nine blighted stems of *H. polyrhizus*. Based on morphological characteristics, DNA sequences and phylogeny (ITS, TEF1-α, and β-tubulin), the fungal isolates were identified as *Diaporthe arecae, D. eugeniae*, *D. hongkongensis, D. phaseolorum*, and *D. tectonendophytica*. Six isolates recovered from the Cameron Highlands, Pahang belonged to *D. eugeniae* (DF1 and DF3), *D. hongkongensis* (DF9), *D. phaseolorum* (DF2 and DF12), and *D. tectonendophytica* (DF7), whereas three isolates from Bukit Kor, Terengganu were recognized as *D. arecae* (DFP3), *D. eugeniae* (DFP4), and *D. tectonendophytica* (DFP2). *Diaporthe eugeniae* and *D. tectonendophytica* were found in both Pahang and Terengganu, *D. phaseolorum* and *D. hongkongensis* in Pahang, whereas *D. arecae* only in Terengganu. The role of the *Diaporthe* isolates in causing stem gray blight of *H. polyrhizus* was confirmed. To date, only *D. phaseolorum* has been previously reported on *Hylocereus undatus*. This is the first report on *D. arecae, D. eugeniae, D. hongkongensis, D. phaseolorum,* and *D. tectonendophytica* causing stem gray blight of *H. polyrhizus* worldwide.

## Introduction

Red-fleshed dragon fruit (*Hylocereus polyrhizus*) is one of the most highly demand varieties, grown in Malaysia owing to its nutritional value and attractive color. It belongs to the Cactaceae family. This exotic fruit is locally known as “buah naga” or “buah mata naga”^1^. It is also known as pitaya, strawberry pear, and night-blooming cereus^2^. In 1999, dragon fruit was first introduced in Setiawan, Perak, and Kuala Pilah, Negeri Sembilan, Malaysia. The fruit was named “dragon fruit” owing to the dragon-like scales or bracts on its surface^3^. Aside from having an attractive color and a pleasant taste, it is considered as a healthy fruit containing excessive amounts of vitamin C and water-soluble fiber^4^.

Like other fruit crops in Malaysia, dragon fruit has been infected with a number of fungal diseases, thus jeopardizing its future. Several cases of fungal attacks on dragon fruit have been documented worldwide, namely, *Alternaria* sp.^5^, *Bipolaris cactivora*^6^, *Botryosphaeria dothidea*^7^, *Colletotrichum gloeosporioides*^8^, *Colletotrichum siamense*^9,10^, and *Colletotrichum truncatum*^11^, *Diaporthe phaseolorum*^12^, *Fusarium oxysporum*^13^, and *Fusarium solani*^14^, *Gilbertella persicaria*^15^, *Lasiodiplodia theobromae*^16^, *Monilinia fructicola*^17^, *Neoscytalidium dimidiatum*^18,19^, *Nigrospora sphaerica*^20^, and *Sclerotium rolfsii*^21^. In Malaysia, previous studies have identified a range of fungal diseases on dragon fruit, including anthracnose^22–24^, stem necrosis^25,26^, stem rot^27,28^, stem blight^29^, and reddish-brown spot^30^.

Dragon fruits with stem gray blight were found in two locations, namely, Bukit Kor, Terengganu, Malaysia, and the Cameron Highlands, Pahang, Malaysia, in November 2017 and July 2018, respectively. These fruits exhibited irregular gray chlorotic lesion on the stem surface and black pycnidia on the infected part. In both locations, of the 50 dragon fruit plants, 20 (40% disease incidence) had been infected with the stem gray blight disease, which may result in its reduced production. This study could provide insights into the management of plant diseases. This study aimed to identify the causal pathogen of the stem gray blight of *H. polyrhizus* via morphological, molecular, and pathogenicity analyses.

## Results

### Fungal isolation and morphological identification

**Figure 1:**
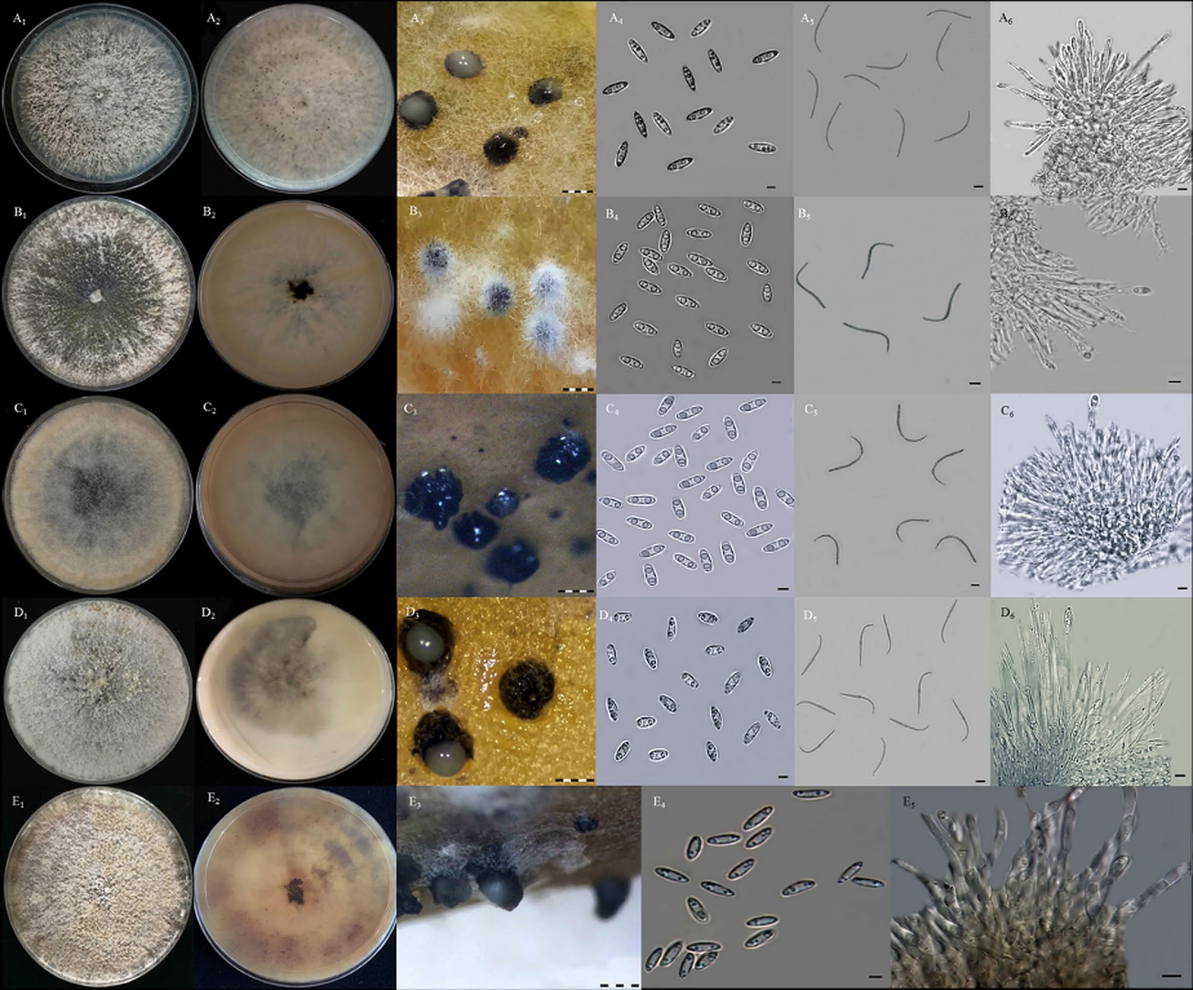
Morphological characteristics of *Diaporthe* species isolated from stem gray blight of *H. polyrhizus*. Group 1 (**A**_**1**_–**A**_**6**_): (**A**_**1**_) colony appearance, (**A**_**2**_) pigmentation, (**A**_**3**_) pycnidial conidiomata, (**A**_**4**_) α-conidia, (**A**_**5**_) β-conidia, (**A**_**6**_) conidiogenous cell for α-conidia; Group 2 (**B**_**1**_–**B**_**6**_): (**B**_**1**_) colony appearance, (**B**_**2**_) pigmentation, (**B**_**3**_) pycnidial conidiomata, (**B**_**4**_) α-conidia, (**B**_**5**_) β-conidia, (**B**_**6**_) conidiogenous cell for α-conidia; Group 3 (**C**_**1**_–**C**_**6**_): (**C**_**1**_) colony appearance, (**C**_**2**_) pigmentation, (**C**_**3**_) pycnidial conidiomata, (**C**_**4**_) α-conidia, (**C**_**5**_) β-conidia, (**C**_**6**_) conidiogenous cell for α-conidia; Group 4 (**D**_**1**_–**D**_**6**_): (**D**_**1**_) colony appearance, (**D**_**2**_) pigmentation, (**D**_**3**_) pycnidial conidiomata, (**D**_**4**_) α-conidia, (**D**_**5**_) β-conidia, (**D**_**6**_) conidiogenous cell for α-conidia; Group 5 (**E**_**1**_–**E**_**5**_): (**E**_**1**_) colony appearance, (**E**_**2**_) pigmentation, (**E**_**3**_) pycnidial conidiomata, (**E**_**4**_) α-conidia, (**E**_**5**_) conidiogenous cell for α-conidia. Scale bar: **A**_**3**_–**E**_**3**_ = 1000 µm; **A**_**4**_–**A**_**6**_, **B**_**4**_–**B**_**6**_, **C**_**4**_–**C**_**6**_, **D**_**4**_–**D**_**6**_, **E**_**4**_–**E**_**5**_: 0.5 µm.

A total of nine fungal isolates were recovered from nine gray blighted stems obtained from the different plants of *H. polyrhizus.* Of these, three isolates (DFP2, DFP3, and DFP4) were recovered from Bukit Kor, Terengganu and six isolates (DF1, DF2, DF3, DF7, DF9, and DF12) from the Cameron Highlands, Pahang, Malaysia. A species or isolate was recovered from a single lesion. In general, the fungal isolates produced whitish, grayish, or brownish colonies on potato dextrose agar (PDA) plates. Two types of conidia, namely, α- and β-conidia, were produced from the formation of pycnidial conidiomata on carnation leaf agar (CLA). α-conidia were characterized as aseptate, hyaline, and fusiform with bi- or multi-guttulate, meanwhile, β-conidia were characterized as aseptate, hyaline, filiform, straight, or more often hamate, and lack guttule. The conidiogenous cells of α-conidia were phialidic, cylindrical, terminal, hyaline, and slightly tapered toward the end. However, in this study, the structure of the conidiogenous cells for β-conidia was not observed. Conidiophore was characterized as hyaline, branched, multiseptate, and filiform. Based on the described characteristics, the fungal isolates were tentatively identified as *Diaporthe* species. By sorting their morphological similarities and differences, the fungal isolates were classified into five groups of *Diaporthe* species (Fig. 1, Table 1).

**Table 1:** Morphological characteristics of five different groups of *Diaporthe* isolates recovered from stem gray blight of *H. polyrhizus.*

Group/isolate	Morphological characteristics					
	Colony on PDA	Pycnidial conidiomata on CLA	^A^α-conidia	^A^β-conidia	Conidiophore of α-conidia	Conidiogenous cell of α-conidia
Group 1 DF1 DF3 DFP4	Abundant and whitish-brown aerial mycelia Whitish-brown on the lower surface	Black and globose Presence of whitish conidial masses exudation	Fusiform, slightly tapered end, aseptate, and hyaline Conidia with size of 6.33 ± 0.68^a^ × 1.98 ± 0.25^a^ µm Bi/multi-guttulate with size of 0.41 ± 0.07^a^ µm	Filiform to hamate, aseptate, and hyaline Conidia with size of 24.57 ± 2.77^b^ × 1.33 ± 0.29^a^ µm	Hyaline, branched, and straight to slightly curve	Cylindrical phialides, terminal, hyaline, and slightly tapered towards end
Group 2 DF2 DF12	Cottony and whitish aerial mycelium Brownish-white on the lower surface	Black and globose	Ovoid with bluntly rounded base end, aseptate, and hyaline Conidia with size of 6.43 ± 0.55^a^ × 2.38 ± 0.21^b^ µm Bi-guttulate with size of 1.53 ± 0.17^c^ µm	Filiform to hamate, aseptate, and hyaline Conidia with size of 17.34 ± 2.17^a^ × 1.49 ± 0.34^a^ µm	Hyaline, branched, and straight to slightly curve	Cylindrical phialides, terminal, hyaline, and slightly tapered towards end
Group 3 DFP2 DF7	Cottony and brownish-white aerial mycelia Brownish colour on the lower surface	Black and globose	Fusoid with bluntly rounded on both ends, aseptate, and hyaline Conidia with size of 6.00 ± 0.81^a^ × 2.39 ± 0.35^b^ µm Bi-guttulate with size of 1.55 ± 0.13^c^ µm	Filiform to hamate, aseptate, and hyaline Conidia with size of 16.29 ± 4.22^a^ × 1.20 ± 0.44^a^ µm	Hyaline, branched, and straight to slightly curve	Cylindrical phialides, terminal, hyaline, and slightly tapered towards end
Group 4 DF9	Cottony and grayish-white aerial mycelium Whitish with gray-patches on the lower surface	Black and globose Presence of whitish conidial masses exudation	Fusiform with tapering towards both ends, aseptate, and hyaline Conidia with size of 6.28 ± 0.64^a^ × 2.57 ± 0.22^b^ µm Bi-guttulate with size of 0.58 ± 0.07^b^ µm	Filiform to hamate, aseptate, and hyaline Conidia with size of 18.29 ± 2.26^a^ × 1.21 ± 0.26^a^ µm	Hyaline, branched, and straight to slightly curve	Cylindrical phialides, terminal, hyaline, and slightly tapered towards end
Group 5 DFP3	Cottony and brownish-white aerial mycelia Yellowish-brown on the lower surface	Black and globose Presence of whitish conidial masses exudation	Fusiform with slightly pointed ends, aseptate, and hyaline Conidia with size of 7.06 ± 0.55^b^ × 2.47 ± 0.34^b^ µm Bi-guttulate with size of 0.40 ± 0.07^a^ µm	Not observed	Hyaline, branched, and straight to slightly curve	Cylindrical phialides, terminal, hyaline, and slightly tapered towards end

^A^Means ± standard deviation followed by different letters within the column are significantly different (*p* < 0.05) according to Tukey’s test.

### Molecular identification and phylogenetic analysis

**Figure 2:**
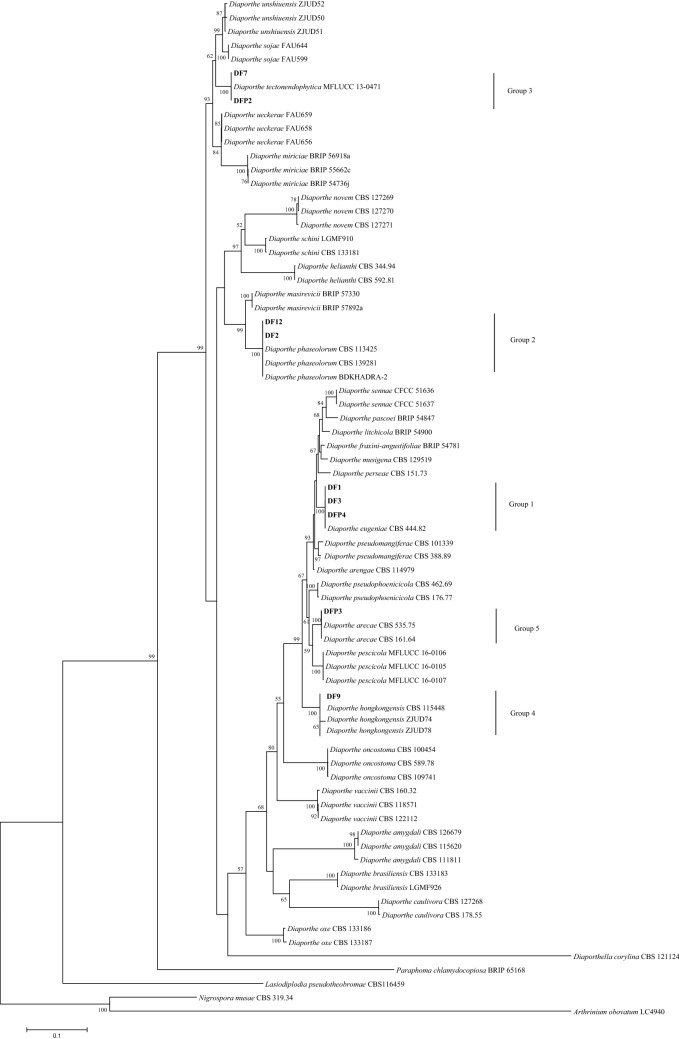
Maximum-likelihood tree of *Diaporthe* species isolated from stem gray blight of *H. polyrhizus* based on combined dataset of ITS, TEF1-α, and β-tubulin using Tamura and Nei model with 1000 bootstrap replications. Isolates of the present study are presented in bold and other fungal genera are used as an outgroup*.* Bootstrap values are shown at the nodes and the scale bar indicates the number of substitutions per position.

The comparison of DNA sequences based on ITS, TEF1-α, and β-tubulin demonstrated that the isolates were similar to the reference sequences of *D. eugeniae, D. phaseolorum, D. tectonendophytica, D. hongkongensis*, and *D. arecae* from the Genbank database. The phylogenetic trees generated from each single gene had the same topology as the tree generated from the combined genes of ITS, TEF1-α, and β-tubulin (Fig. 2) (Supplementary Information). The groupings of each single tree demonstrated that all the isolates were clustered in the same clades as their respective species of *Diaporthe* (*D. eugeniae, D. phaseolorum, D. tectonendophytica, D. hongkongensis,* and *D. arecae*). Isolates DF1, DF3, and DFP4 were grouped with *D. eugeniae* CBS 444.82; isolates DF2 and DF12 with *D. phaseolorum* CBS113425 and BDKHADRA-2; isolates DFP2 and DF7 with *D. tectonendophytica* MFLUCC 13-0471; and isolates DF9 and DFP3 with *D. hongkongensis* CBS 115448 and *D. arecae* CBS 161.64, respectively. The result of the phylogenetic analysis was in accordance with the molecular identification based on DNA sequences [Basic Local Alignment Search (BLAST)], thus resolving the morphological identification. The isolates from group 1 were confirmed to be *D. eugeniae*, group 2 was *D. phaseolorum*, group 3 was *D. tectonendophytica,* group 4 was *D. hongkongensis,* and group 5 was *D. arecae.* The combined sequence matrix and phylogenetic tree were deposited in TreeBASE (http://purl.org/phylo/treebase/phylows/study/TB2:S27649).

### Pathogenicity test and comparative aggressiveness among *Diaporthe* isolates

**Figure 3:**
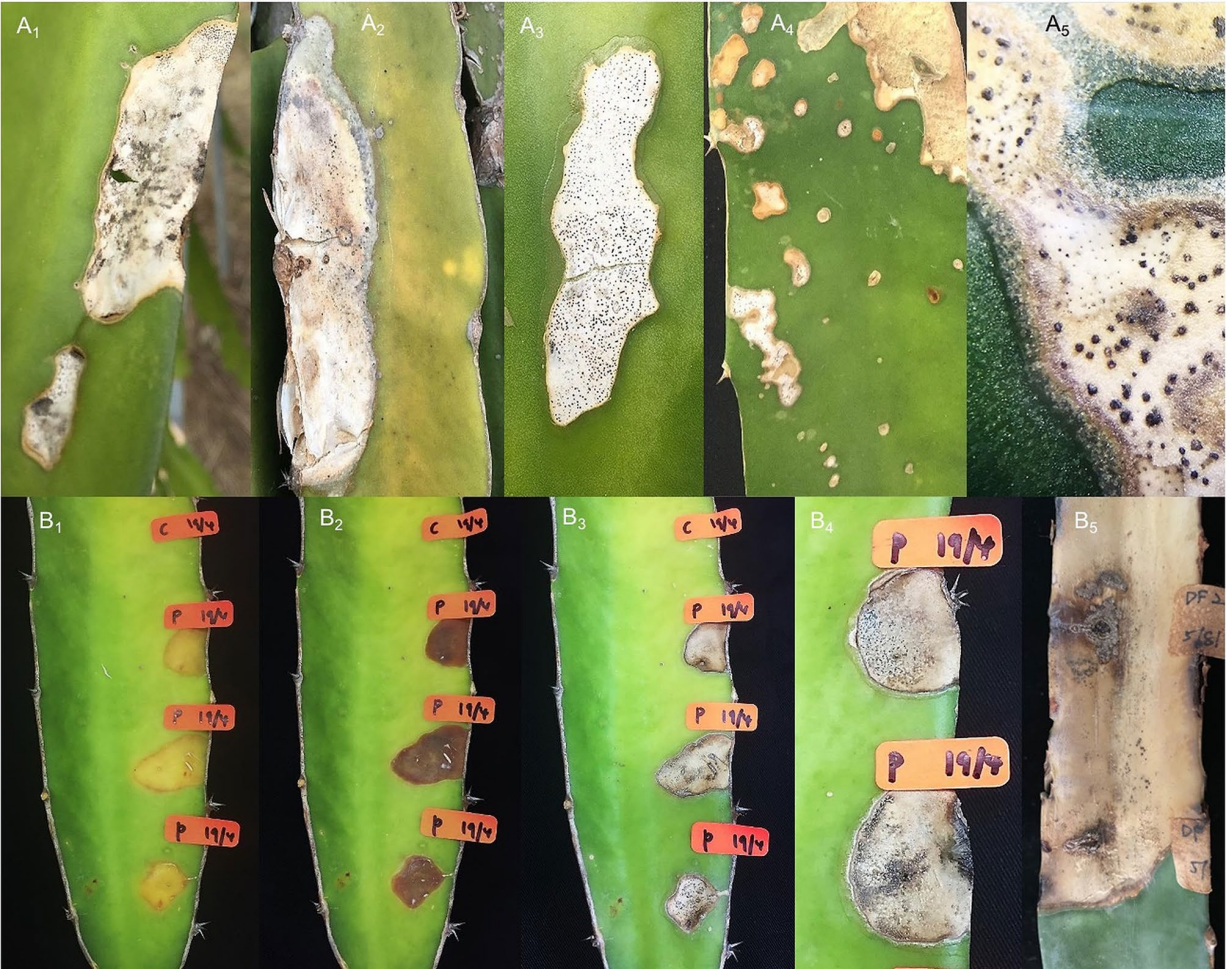
Stem gray blight of *H. polyrhizus*. (**A**_**1**_–**A**_**5**_) Disease symptoms observed in the fields. (**B**_**1**_) After 2 days of inoculation, irregular yellowish lesions surrounded by reddish borders appeared. (**B**_**2**_) The lesions became sunken and turned darker. (**B**_**3**_) The lesions apparently dry and turned to gray. (**B**_**4**_–**B**_**5**_) At later stage, the lesions expanded resulting in the appearance of blighted stem with formation of tiny black pycnidia. C denotes control and P represents treatment.

The result of pathogenicity test indicated that all isolates of the *Diaporthe* species recovered from the stem gray blight of *H. polyrhizus* were pathogenic, exhibiting similar symptoms to those in the field (Fig. 3A_1_–A_5_). The tested isolates showed typical symptoms of gray blight on the inoculated stems of *H. polyrhizus*. Initially, irregular yellowish lesion surrounded by reddish border appeared on the wounded point (Fig. 3B_1_), which gradually turned into a dark-brown sunken lesion and demonstrated dampening (Fig. 3B_2_). As the disease progressed, the lesion became apparently dry and turned gray (Fig. 3B_3_). Then, it expanded periodically, and tiny black pycnidia appeared on the area of the lesion (Fig. 3B_4_–B_5_). No symptoms developed on the control points.

Isolate DF1 (*D. eugeniae*) recorded the highest lesion length (10.25 ± 0.35 cm), whereas isolate DFP3 (*D. arecae*) had the lowest (3.25 ± 0.35 cm) (Table 2). The means of the length lesion of the tested isolates were significantly different compared with the control at *p* < 0.05. The tested isolates of *Diaporthe* exhibited variability in length lesion after 3 weeks of inoculation on the stems of *H. polyrhizus*. The same *Diaporthe* species were reisolated from the symptomatic inoculated stems of *H. polyrhizus*, and their identities were reconfirmed by comparing the macroscopic and microscopic characteristics with the original cultures, thus fulfilling Koch’s postulates.

**Table 2:** Lesion length recorded by *Diaporthe* isolates after 3 weeks of inoculation on stems of *H. polyrhizus.*

Species	Isolate	^A^Lesion length (cm)
*D. eugeniae*	DF1	10.25 ± 0.35^e^
	DF3	5.50 ± 0.70^c^
	DFP4	5.10 ± 0.84^bc^
*D. phaseolorum*	DF2	7.50 ± 0.00^d^
	DF12	7.75 ± 0.35^d^
*D. tectonendophytica*	DF7	8.25 ± 0.35^d^
	DFP2	3.45 ± 0.70^ab^
*D. hongkongensis*	DF9	3.50 ± 0.00^ab^
*D. arecae*	DFP3	3.25 ± 0.35^a^
Control		0.00 ± 0.00^f^

^A^Mean ± standard deviation followed by different letters within the column is significantly different (*p* < 0.05) according to Tukey’s test.

## Discussion

The present study reported on stem gray blight, which is a new emerging disease infecting *H. polyrhizus* plantations in Malaysia. The five species of *Diaporthe*, namely, *D. eugeniae* (group 1), *D. phaseolorum* (group 2), *D. tectonendophytica* (group 3), *D. hongkongensis* (group 4), and *D. arecae* (group 5)*,* were identified to be the causal agents of the disease. The *Diaporthe* species may act as a plant pathogen or a saprophyte or an endophytic symbiont^31–34^, however, several studies have reported that it is the genus responsible for multiple destructive diseases, such as root and fruit rots, dieback, stem cankers, leaf spots, leaf and pod blights, and seed decay^31,33,35–39^.

A total of nine *Diaporthe* isolates were recovered from the blighted stem of *H. polyrhizus*. Based on their morphological characteristics, all the isolates produced both α-conidia and β-conidia, except for the *D. arecae* isolate, of which β-conidia was not observed. α- and β-conidia are the key characteristics for the identification of *Diaporthe*^33,40^. The formation of β-conidia can sometimes be rare or absent in certain species of *Diaporthe*^41^. According to Tuset and Portilla^42^ and Diogo et al*.*^43^, for some *Diaporthe* species (e.g. *Phomopsis amygdali*), the formation of β-conidia can only be observed in pycnidia on the host but not in pycnidia in the culture plate.

Based on the similarities and differences of their macroscopic and microscopic characteristics, the isolates were assigned to five different groups. Among the groups, significant differences were observed in the number of α-conidia guttules and their size (Table 1). Gomes et al*.*^34^ revealed that both characteristics can be varied among the *Diaporthe* species. The isolates from group 1 (*D. eugeniae*) tended to produce bi- and multi-guttules, whereas the other isolates only produced bi-guttules of α-conidia. The size of the guttules of α-conidia varied among the groups. The isolates from groups 1 and 5 (*D. eugeniae* and *D. arecae*) produced significantly smaller guttules compared with those produced by isolates from groups 2, 3, and 5 (*D. phaseolorum*, *D. tectonendophytica*, and *D. hongkongensis*) (Table 1). The guttule is defined as a small drop or particle in a spore resembling a nucleus^44^. Moreover, the morphology of α-conidia of the *D. eugeniae*, *D. hongkongensis*, and *D. arecae* isolates was tapered toward the ends compared with the *D. phaseolorum* and *D. tectonendophytica* isolates, the ends of which were bluntly rounded (Fig. 1). This finding was in agreement with those of Santos et al*.*^38^, Dissanayake et al*.*^45^, Doilom et al*.*^46^, and Lim et al*.*^47^. A significant difference was also observed in the length of β-conidia, of which the *D. eugeniae* isolates produced longer β-conidia than other isolates from different groups. Conidial mass exudation can be observed in the isolates of *D. eugeniae, D. hongkongensis*, and *D. arecae*. Contrarily, it was not observed in the isolates of *D. phaseolorum* and *D. tectonendophytica*. According to Machowicz-Stefaniak et al*.*^48^, the *Diaporthe* species require temperatures ranging from 22 to 28 °C for the optimal growth, sporulation, and rate of conidia release of conidiomata. As applied in the present study, the addition of carnation leaves to the growing medium as substrates has been recommended to improve the sporulation of the *Diaporthe* species^49,50^.

Aside from the microscopic characteristic, the cultural characteristics of all isolates in this study also varied among the groups. The color of the colonies ranged from whitish, grayish, brownish, to olive green. Due to this inconsistency, cultural characteristic is commonly considered as a less important criterion in distinguishing species within *Diaporthe* as it can be influenced by several environmental factors, such as light and temperature^34^. Based on the results obtained, morphological characteristics alone were insufficient to identify all the isolates up to the species level due to the complexity of the genus. This finding was in agreement with that of Lim et al*.*^47^ who revealed that the morphological method alone is not informative for the species identification of *Diaporthe* due to pleomorphism and overlapping characteristics^43,51,52^.

With the advances in molecular techniques, DNA sequences and multigene phylogenetic analysis of ITS, TEF1-α, and β-tubulin were employed to support the morphological identification of the *Diaporthe* isolates in this study. The result of the BLAST search and phylogenetic inference indicated that the use of all the three genes resolved identification of the *Diaporthe* isolates. Aside from the present study, ITS, TEF1-α, and β-tubulin were extensively applied to delineate species within *Diaporthe*^46,53,54^. The ITS region served as an identification guide for the *Diaporthe* species^33^. It was also considered as a fungal barcode in distinguishing genera and species owing to its easy amplification and ability to provide preliminary screening of fungal classification^55,56^. However, the tree constructed based on ITS sequences alone may be doubtful and not demonstrate clear phylogenetic relationships due to the lack of interspecific variation or even deceptive in some fungi^57^. Thus, TEF1-α and β-tubulin were added to support the phylogenetic analysis of ITS in delimiting the species of the *Diaporthe* isolates. TEF1-α comprises an essential part of the protein translation machinery, and highly informative at the species level; moreover, non-orthologous copies have not been detected in *Diaporthe*^58^. β-tubulin was utilized as an alternative phylogenetic marker to specify *Diaporthe* as it contains fewer ambiguously aligned regions and exhibits less homoplasy among the genus^59^. Collectively, phylogenetic analysis of a combined dataset of ITS, TEF1-α, and β-tubulin was conducted in this study to overcome the ambiguity that could have emerged in the single gene analysis. Santos et al*.*^60^ stated that the combined phylogenetic tree commonly provides a better resolution for the identification of the *Diaporthe* species compared with the single gene analysis.

All the tested isolates of *Diaporthe* exhibited varying lengths of lesion on the inoculated stems of *H. polyrhizus*, of which isolate DF1 (*D. eugeniae*) was found to be the most virulent. The fungus can act as a pathogen or a saprophyte and was reported to cause stem-end rot on mango (*Mangifera indica*)^47^. It also occurs as a saprophyte on cloves (*Eugenia aromatica*)^34^. This study discovered a new host and disease caused by *D. eugeniae*. The association of *D. phaseolorum* with dragon fruit was not new, because recently, this pathogen was reported to cause stem rot on *Hylocereus undatus* in Bangladesh^12^. However, the symptoms described were slightly different from those observed in the present study. It appeared as a yellow spot with a chlorotic halo in the previous report, but in the present study, chlorotic halo was not observed; rather, a reddish border surrounded the lesion. Similarly, gray to black pycnidia were scattered on the surface of the lesion. Aside from the dragon fruit, *D. phaseolorum* was reported as a causal agent of pod and stem blight, stem canker, and seed rot on soybean and trunk disease on grapevine^38,45,61,62^. It was also found to be an endophyte on *Kandelia candel* by Cheng et al*.*^63^.

Similar to *D. eugeniae,* the present study highlighted *H. polyrhizus* as a new host associated with *D. tectonendophytica* as it causes stem gray blight. Contrarily, a study by Doilom et al*.*^46^ demonstrated the role of *D. tectonendophytica* as an endophyte occurring on teak (*Tectona grandis*) in Thailand. The capability of *D. hongkongensis* to act as a pathogen is undeniable as the fungus has been reported to cause severe diseases on a number of host plants, such as stem-end rot on kiwifruit^64^, dieback on grapevine^45^, and shoot canker on pear^65^. Meanwhile, *D. arecae* has been reported to be pathogenic on *M. indica*^47^, *Areca catechu*^34^, and *Citrus*^66^. *D. hongkongensis* and *D. arecae* were first reported on *H. polyrhizus* worldwide especially in Malaysia.

The occurrence of the disease in two different locations in Malaysia indicates its possibility to spread worldwide. Aside from *Diaporthe*, dragon fruits in Malaysia also suffer from multiple diseases caused by other fungi. Among these diseases are anthracnose caused by *C. gloeosporioides*^22,23^ and *C. truncatum*^24^; stem necrosis by *Curvularia lunata*^25^; stem canker by *N. dimidiatum*^26^; stem rot by *Fusarium proliferatum*^27^ and *Fusarium fujikuroi*^28^; reddish brown spot by *Nigrospora lacticolonia* and *N. sphaerica*^30^; and stem blight by *F. oxysporum*^29^.

This study provides overview of the five different species of *Diaporthe* causing stem gray blight on *H. polyrhizus* in Malaysia. It improves our knowledge on the symptomatology of the disease and identity of the pathogens through morphological and molecular analyses. The findings may be essential to strategize effective disease management for stem gray blight on *H. polyrhizus* and for quarantine restrictions.

## Materials and methods

### Fungal isolation

In November 2017 and July 2018, nine gray blighted stems from the different plants of *H. polyrhizus* were collected from Bukit Kor, Terengganu, Malaysia, and the Cameron Highlands, Pahang, Malaysia. The symptomatic samples were brought back to the laboratory for isolation. One lesion per stem exhibiting the same symptom was selected for fungal isolation. The lesion consisting of diseased and healthy parts was excised (1.5 cm^2^) and surface-sterilized with 70% ethanol for 3 min. Then, the samples were soaked in 10% sodium hypochlorite (1% NaOCl) for 3 min and rinsed with sterile distilled water three times consecutively for 1 min each. The sterilized samples were air-dried on the sterile filter papers before being transferred to PDA plates. The inoculated plates were incubated at 25 °C ± 2 °C for 2 to 3 days. Pure cultures of fungal isolates were obtained via hyphal tip isolation and were used for morphological and molecular analyses.

### Morphological identification

Each fungal isolate obtained was cultured on PDA and incubated at 25 °C ± 2 °C for 7 days. Macroscopic characteristics, such as colony appearance and pigmentation, were recorded. CLA was utilized to induce the formation of pycnidial conidiomata, and the inoculated plates were incubated at 25 °C ± 2 °C for 7 days. The morphology of α- and β-conidia was observed from the pycnidial conidiomata. The other microscopic characteristics observed were conidiophores and conidiogenous cells. The length and width of 30 randomly selected conidia and the size of the guttules of 30 randomly selected α-conidia were measured and recorded. The differences in the length and width of conidia and the size of the guttules of α-conidia were evaluated via one-way ANOVA. In addition, the means of both parameters were compared via Tukey’s test (*p* < 0.05) using the IBM SPSS Statistics software version 24.

### Molecular identification and phylogenetic analysis

The identity of all the fungal isolates was further confirmed by molecular characterization. The isolates were grown in potato dextrose broth (PDB) and incubated at 25 °C ± 2 °C for 7 days. Fungal mycelia from PDB were homogenized under liquid nitrogen to obtain fine powder. A total of 60 mg fine powder was transferred into a 1.5 mL microcentrifuge tube, and the genomic DNA of the fungal isolates was extracted using the Invisorb Spin Plant Mini Kit (Stratec Biomedical AG, Birkenfeld, Germany), following the manufacturer’s protocols. The primers of ITS5/ITS4^67^, EF1-728/EF1-986^68^, and BT2a/BT2b^69^ were used for the amplification of ITS, TEF1-α, and β-tubulin, respectively. A total of 50 µL reaction mixture was prepared, which contained 8 µL of green buffer (Promega, USA), 8 µL of MgCl_2_ (Promega, USA), 1 µL of deoxynucleotide triphosphate polymerase (dNTP) (Promega, USA), 8 µL of each primer (Promega, USA), 0.3 µL of Taq polymerase (Promega, USA), 1 µL of genomic DNA, and sterile distilled water. Polymerase chain reaction (PCR) was performed using MyCycler Thermal Cycler (BioRad, Hercules, USA) under the following conditions: initial denaturation at 95 °C for 4 min, followed by 35 cycles of denaturation at 95 °C for 35 s, annealing at 54 °C (ITS)/57 °C (TEF1-α)/58 °C (β-tubulin) for 1 min, extension at 72 °C for 90 s, and final extension at 72 °C for 10 min. The PCR product was separated by running it in 1.0% agarose gel (Promega, USA) stained with HealthView Nucleic Acid Stain (Genomics, Taiwan) at 90 V and 400 mA for 90 min. The 100 bp DNA ladder (Thermo Scientific, USA) was used as a marker to estimate the size of the amplified PCR products. The PCR products were sent to a service provider (First BASE Laboratories Sdn Bhd, Seri Kembangan, Malaysia) for DNA sequencing.

The obtained sequences were aligned using the Molecular Evolutionary Genetic Analysis software (MEGA7)^70^. After pairwise alignment, the BLAST algorithm (https://blast.ncbi.nlm.nih.gov/Blast.cgi) was used to compare the generated consensus sequences with other sequences in the GenBank database. The sequences obtained were deposited in the GenBank database.

The isolates in the present study and reference sequences used in the phylogenetic analysis are presented in Table 3. Multiple sequence alignments of fungal isolates and reference isolates were generated using the MEGA7 software. Phylogenetic analysis was conducted using the maximum likelihood (ML) method in MEGA7. The Tamura-Nei model^71^ was used to generate the ML trees based on a single and combined genes of ITS, TEF1-α, and β-tubulin with 1000 bootstrap replications^72^.

**Table 3:** Isolates in the present study and reference isolates used in the phylogenetic analysis.

Species	Isolate	Host	Locality	GenBank accession no.			References
				ITS	TEF1-α	β-tubulin	
*D. amygdali*	CBS 126679^EP^	*Prunus dulcis*	Portugal	KC343022	KC343748	KC343990	Gomes et al*.*^34^
*D. amygdali*	CBS 111811	*Vitis vinifera*	South Africa	KC343019	KC343745	KC343987	Gomes et al*.*^34^
*D. amygdali*	CBS 115620	*Prunus persica*	USA	KC343020	KC343746	KC343988	Gomes et al*.*^34^
*D. arecae*	CBS 161.64^EI^	*Arecae catechu*	India	KC343032	KC343758	KC344000	Gomes et al*.*^34^
*D. arecae*	CBS 535.75	*Citrus* sp.	Suriname	KC343033	KC343759	KC344001	Gomes et al*.*^34^
*Diaporthe* sp. (Group 5)	DFP3	*Hylocereus polyrhizus*	Bukit Kor, Terengganu, Malaysia	MN862382	MN889938	MN889947	This study
*D. arengae*	CBS 114979^ET^	*Arenga engleri*	Hong Kong	KC343034	KC343760	KC344002	Gomes et al*.*^34^
*D. brasiliensis*	CBS 133183^ET^	*Aspidosperma tomentosum*	Brazil	KC343042	KC343768	KC344010	Gomes et al*.*^34^
*D. brasiliensis*	LGMF 926	*Aspidosperma tomentosum*	Brazil	KC343043	KC343769	KC344011	Gomes et al*.*^34^
*D. caulivora*	CBS 127268^EN^	*Glycine max*	Croatia	KC343045	KC343771	KC344013	Gomes et al*.*^34^
*D. caulivora*	CBS 178.55	*Glycine soja*	Canada	KC343046	KC343772	KC344014	Gomes et al*.*^34^
*D. eugeniae*	CBS 444.82	*Eugenia aromatica*	Indonesia	KC343098	KC343824	KC344066	Gomes et al*.*^34^
*Diaporthe* sp. (Group 1)	DF1	*Hylocereus polyrhizus*	Cameron Highlands, Pahang, Malaysia	MN862375	MN889932	MN889940	This study
*Diaporthe* sp. (Group 1)	DF3	*Hylocereus polyrhizus*	Cameron Highlands, Pahang, Malaysia	MN862377	MN889935	MN889944	This study
*Diaporthe* sp. (Group 1)	DFP4	*Hylocereus polyrhizus*	Bukit Kor, Terengganu, Malaysia	MN862383	MN889939	MN889948	This study
*D. fraxini-angustifoliae*	BRIP 54781^EI^	*Fraxinus angustifolia*	Australia	JX862528	JX862534	KF170920	Tan et al*.*^73^
*D. helianthi*	CBS 592.81^ET^	*Helianthus annuus*	Serbia	KC343115	KC343841	KC344083	Gomes et al*.*^34^
*D. helianthi*	CBS 344.94	*Helianthus annuus*	–	KC343114	KC343840	KC344082	Gomes et al*.*^34^
*D. hongkongensis*	CBS 115448^ET^	*Dichroa febrifuga*	Hong Kong	KC343119	KC343845	KC344087	Gomes et al*.*^34^
*D. hongkongensis*	ZJUD74	*Citrus unshiu*	China	KJ490609	KJ490488	KJ490430	Huang et al*.*^66^
*D. hongkongensis*	ZJUD78	*Citrus unshiu*	China	KJ490613	KJ490492	KJ490434	Huang et al*.*^66^
*Diaporthe* sp. (Group 4)	DF9	*Hylocereus polyrhizus*	Cameron Highlands, Pahang, Malaysia	MN862379	MN889933	MN889941	This study
*D. litchicola*	BRIP 54900^EH^	*Litchi chinensis*	Australia	JX862533	JX862539	KF170925	Tan et al.^73^
*D. masirevicii*	BRIP 57892a^EH^	*Helianthus annuus*	Australia	KJ197276	KJ197239	KJ197257	Thompson et al*.*^74^
*D. masirevicii*	BRIP 57330	*Chrysanthemoides monilifera*	Australia	KJ197275	KJ197237	KJ197255	Thompson et al*.*^74^
*D. miriciae*	BRIP 54736j^EH^	*Helianthus annuus*	Australia	KJ197282	KJ197244	KJ197262	Thompson et al*.*^74^
*D. miriciae*	BRIP 55662c	*Glycine max*	Australia	KJ197283	KJ197245	KJ197263	Thompson et al*.*^74^
*D. miriciae*	BRIP 56918a	*Vigna radiata*	Australia	KJ197284	KJ197246	KJ197264	Thompson et al*.*^74^
*D. musigena*	CBS 129519^ET^	*Musa* sp.	Australia	KC343143	KC343869	KC344111	Gomes et al*.*^34^
*D. novem*	CBS 127270^ET^	*Glycine max*	Croatia	KC343156	KC343882	KC344124	Gomes et al*.*^34^
*D. novem*	CBS 127269	*Glycine max*	Croatia	KC343155	KC343881	KC344123	Gomes et al*.*^34^
*D. novem*	CBS 127271	*Glycine max*	Croatia	KC343157	KC343883	KC344125	Gomes et al*.*^34^
*D. oncostoma*	CBS 589.78	*Robinia pseudoacacia*	France	KC343162	KC343888	KC344130	Gomes et al*.*^34^
*D. oncostoma*	CBS 100454	*Robinia pseudoacacia*	Germany	KC343160	KC343886	KC344128	Gomes et al*.*^34^
*D. oncostoma*	CBS 109741	*Robinia pseudoacacia*	Russia	KC343161	KC343887	KC344129	Gomes et al*.*^34^
*D. oxe*	CBS 133186^ET^	*Maytenus ilicifolia*	Brazil	KC343164	KC343890	KC344132	Gomes et al*.*^34^
*D. oxe*	CBS 133187	*Maytenus ilicifolia*	Brazil	KC343165	KC343891	KC344133	Gomes et al*.*^34^
*D. pascoei*	BRIP 54847^EI^	*Perseae americana*	Australia	JX862532	JX862538	KF170924	Tan et al*.*^73^
*D. perseae*	CBS 151.73	*Perseae americana*	Netherlands	KC343173	KC343899	KC344141	Gomes et al*.*^34^
*D. pescicola*	MFLUCC 16-0105^EH^	*Prunus persica*	China	KU557555	KU557623	KU557579	Dissanayake et al*.*^75^
*D. pescicola*	MFLUCC 16-0106	*Prunus persica*	China	KU557556	KU557624	KU557580	Dissanayake et al*.*^75^
*D. pescicola*	MFLUCC 16-0107	*Prunus persica*	China	KU557557	KU557625	KU557581	Dissanayake et al*.*^75^
*D. phaseolorum*	CBS 139281^EP^	*Phaseolus vulgaris*	USA	KJ590738	KJ590739	KJ610893	Udayanga et al*.*^76^
*D. phaseolorum*	CBS 113425	*Olearia* cf. *rani*	New Zealand	KC343174	KC343900	KC344142	Gomes et al*.*^34^
*D. phaseolorum*	BDKHADRA-2	*Hylocereus undatus*	Bangladesh	MH714560	KC343902	KC344144	Karim et al*.*^12^
*Diaporthe* sp. (Group 2)	DF2	*Hylocereus polyrhizus*	Cameron Highlands, Pahang, Malaysia	MN862376	MN889931	MN889942	This study
*Diaporthe* sp. (Group 2)	DF12	*Hylocereus polyrhizus*	Cameron Highlands, Pahang, Malaysia	MN862380	MN889936	MN889945	This study
*D. pseudomangiferae*	CBS 101339^ET^	*Mangifera indica*	Dominican Republic	KC343181	KC343907	KC344149	Gomes et al*.*^34^
*D. pseudomangiferae*	CBS 388.89	*Mangifera indica*	Mexico	KC343182	KC343908	KC344150	Gomes et al*.*^34^
*D. pseudophoenicicola*	CBS 462.69^ET^	*Phoenix dactylifera*	Spain	KC343184	KC343910	KC344152	Gomes et al*.*^34^
*D. pseudophoenicicola*	CBS 176.77	*Mangifera indica*	Iraq	KC343183	KC343909	KC344151	Gomes et al*.*^34^
*D. schini*	CBS 133181^ET^	*Schinus terebinthifolius*	Brazil	KC343191	KC343917	KC344159	Gomes et al*.*^34^
*D. schini*	LGMF 910	*Schinus terebinthifolius*	Brazil	KC343192	KC343918	KC344160	Gomes et al*.*^34^
*D. sennae*	CFCC 51636^EH^	*Senna bicapsularis*	China	KY203724	KY228885	KY228891	Yang et al*.*^77^
*D. sennae*	CFCC 51637	*Senna bicapsularis*	China	KY203725	KY228886	KY228892	Yang et al*.*^77^
*D. sojae*	FAU 599^EH^	*Glycine max*	USA	KJ590728	KJ590767	KJ610883	Udayanga et al*.*^76^
*D. sojae*	FAU 644	*Glycine max*	USA	KJ590730	KJ590769	KJ610885	Udayanga et al*.*^76^
*D. tectonendophytica*	MFLUCC 13-0471^EH^	*Tectona grandis*	Thailand	KU712439	KU749367	KU743986	Doilom et al*.*^46^
*Diaporthe* sp. (Group 3)	DF7	*Hylocereus polyrhizus*	Cameron Highlands, Pahang, Malaysia	MN862378	MN889934	MN889943	This study
*Diaporthe* sp. (Group 3)	DFP2	*Hylocereus polyrhizus*	Bukit Kor, Terengganu, Malaysia	MN862381	MN889937	MN889946	This study
*D. ueckerae*	FAU 656^EH^	*Cucumis melo*	USA	KJ590726	KJ590747	KJ610881	Udayanga et al*.*^76^
*D. ueckerae*	FAU 659	*Cucumis melo*	USA	KJ590724	KJ590745	KJ610879	Udayanga et al*.*^76^
*D. ueckerae*	FAU 658	*Cucumis melo*	USA	KJ590725	KJ590746	KJ610880	Udayanga et al*.*^76^
*D. unshiuensis*	ZJUD 52	*Citrus unshiu*	China	KJ490587	KJ490466	KJ490408	Huang et al*.*^66^
*D. unshiuensis*	ZJUD 50	*Citrus japonica*	China	KJ490585	KJ490464	KJ490406	Huang et al*.*^66^
*D. unshiuensis*	ZJUD 51	*Citrus japonica*	China	KJ490586	KJ490465	KJ490407	Huang et al*.*^66^
*D. vaccinii*	CBS 160.32^ET^	*Oxycoccus macrocarpos*	USA	KC343228	KC343954	KC344196	Gomes et al*.*^34^
*D. vaccinii*	CBS 118571	*Vaccinium corymbosum*	USA	KC343223	KC343949	KC344191	Gomes et al*.*^34^
*D. vaccinii*	CBS 122112	*Vaccinium macrocarpon*	USA	KC343224	KC343950	KC344192	Gomes et al*.*^34^
*Diaporthella corylina*	CBS 121124	*Corylus* sp.	China	KC343004	KC343730	KC343972	Gomes et al*.*^34^
*Lasiodiplodia pseudotheobromae*	CBS 116459^ET^	*Gmelina arborea*	Costa Rica	EF622077	EF622057	EU673111	Alves et al*.*^78^
*Nigrospora musae*	CBS 319.34^EH^	*Musa paradisiaca*	Australia	KX986076	KY019419	KY019455	Wang et al*.*^79^
*Arthrinium obovatum*	CGMCC 3.18331^EH^	*Lithocarpus* sp.	China	KY494696	KY705095	KY705166	Wang et al.^80^
*Paraphoma chlamydocopiosa*	BRIP 65168^EH^	*Tanacetum cinerariifolium*	Australia	KU999072	KU999080	KU999084	Moslemi et al.^81^

*EP* ex-epitype culture, *EI* ex-isotype culture, *ET* ex-type culture, *EN* ex-neotype culture, *EH* ex-holotype culture.

### Pathogenicity test

The pathogenicity test was conducted on 18 healthy stems of *H. polyrhizus* for all the obtained fungal isolates. Conidial suspension was prepared by flooding the 7-day-old PDA culture with sterile distilled water, and the concentration was adjusted to 1 × 10^6^ conidia/mL using a hemocytometer (Weber, Teddington, UK). The stems were surface-sterilized with 70% ethanol, and 0.1 mL of conidial suspension was utilized for inoculation using a disposable needle and syringe. Likewise, the control points were treated with sterile distilled water. On each stem, three points were used to inoculate fungal isolate and one point for control. Each fungal isolate was tested in three replicates, and the pathogenicity tests were conducted twice. All the inoculated plants were placed in a plant house in the School of Biological Sciences, USM, and incubated at 26–32 °C for 21 days. The progression of the disease symptom was observed daily. The lesion length was measured and recorded after 3 weeks of inoculation. The differences in the lesion length were evaluated via one-way ANOVA, and the means were compared via Tukey’s test (*p* < 0.05) using the IBM SPSS Statistics software version 24. For the fulfillment of Koch’s postulates, the fungal isolates were reisolated from symptomatic inoculated stems and reidentified by morphological characteristics.

## Supplementary information

Supplementary Information.
