# Supplementary material for: Diaporthe species causing stem gray blight of red-fleshed dragon fruit (Hylocereus polyrhizus) in Malaysia
Source: Sci Rep. 2021 Feb 16;11:3907. doi: 10.1038/s41598-021-83551-z (PMC7887222; doi:10.1038/s41598-021-83551-z)
Supplement: Supplementary file 1 — Supplementary Information. [file 41598_2021_83551_MOESM1_ESM.pdf]

Diaporthe species causing stem gray blight of red-fleshed dragon fruit (_Hylocereus polyrhizus_) in Malaysia

Abd Rahim Huda-Shakirah, Yee Jia Kee, Kak Leong Wong, Latiffah Zakaria, Masratul Hawa Mohd*

School of Biological Sciences, Universiti Sains Malaysia, 11800 Penang, Malaysia

*Corresponding author:

Masratul Hawa Mohd (PhD)

School of Biological Sciences

Universiti Sains Malaysia

11800 Penang

Malaysia

Email: masratulhawa@usm.my

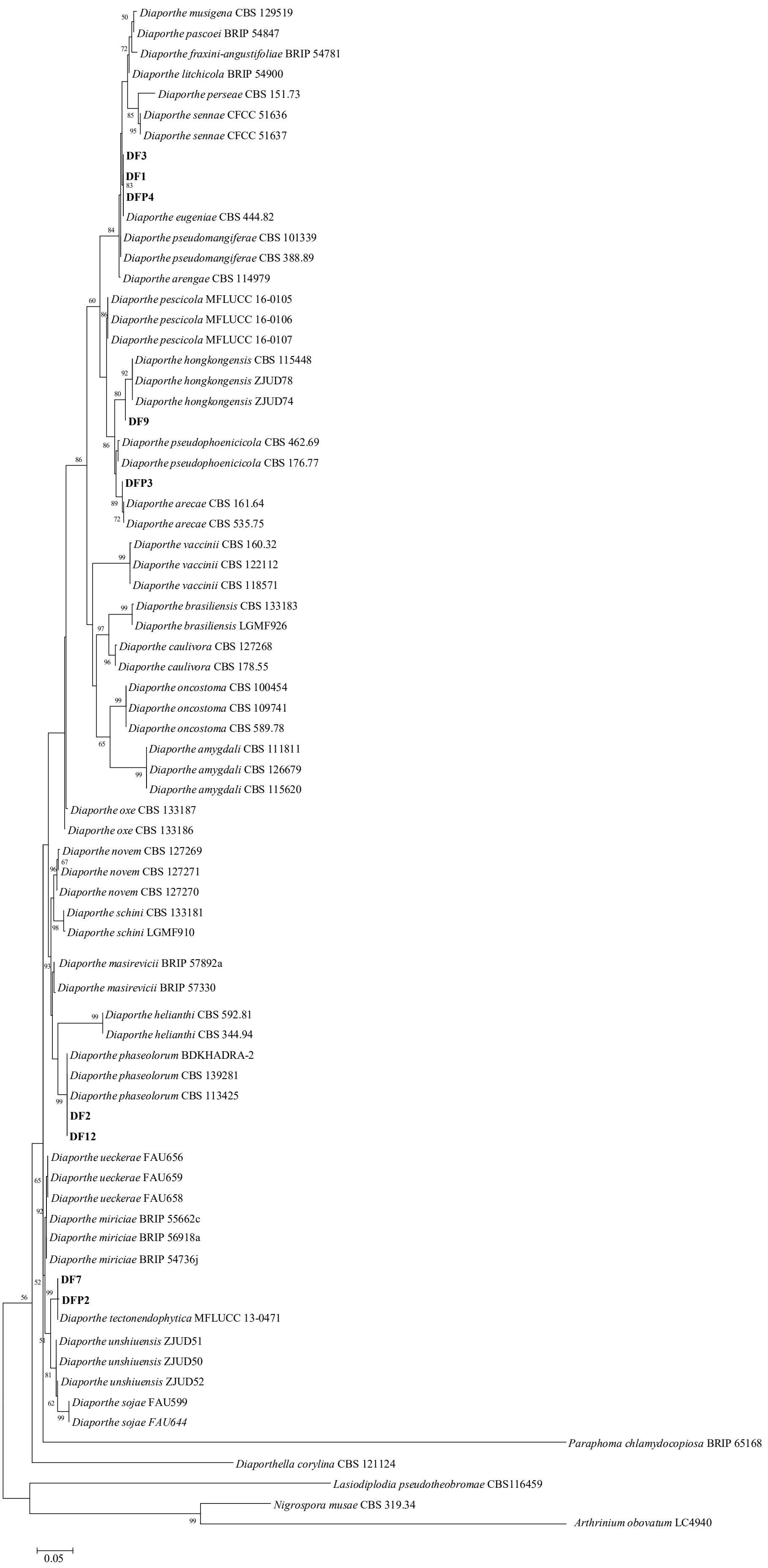
Diaporthe musigena CBS 129519
Diaporthe pascoei BRIP 54847
Diaporthe fraxini-angustifoliae BRIP 54781
Diaporthe litchicola BRIP 54900
Diaporthe perseae CBS 151.73
Diaporthe sennae CFCC 51636
Diaporthe sennae CFCC 51637
DF3
DF1
DFP4
Diaporthe eugeniae CBS 444.82
Diaporthe pseudomangiferae CBS 101339
Diaporthe pseudomangiferae CBS 388.89
Diaporthe arengae CBS 114979
Diaporthe pescicola MFLUCC 16-0105
Diaporthe pescicola MFLUCC 16-0106
Diaporthe pescicola MFLUCC 16-0107
Diaporthe hongkongensis CBS 115448
Diaporthe hongkongensis ZJUD78
Diaporthe hongkongensis ZJUD74
DF9
Diaporthe pseudophoenicicola CBS 462.69
Diaporthe pseudophoenicicola CBS 176.77
DFP3
Diaporthe arecae CBS 161.64
Diaporthe arecae CBS 535.75
Diaporthe vaccinii CBS 160.32
Diaporthe vaccinii CBS 122112
Diaporthe vaccinii CBS 118571
Diaporthe brasiliensis CBS 133183
Diaporthe brasiliensis LGMF926
Diaporthe caulivora CBS 127268
Diaporthe caulivora CBS 178.55
Diaporthe oncostoma CBS 100454
Diaporthe oncostoma CBS 109741
Diaporthe oncostoma CBS 589.78
Diaporthe amygdali CBS 111811
Diaporthe amygdali CBS 126679
Diaporthe amygdali CBS 115620
Diaporthe oxe CBS 133187
Diaporthe oxe CBS 133186
Diaporthe novem CBS 127269
Diaporthe novem CBS 127271
Diaporthe novem CBS 127270
Diaporthe schini CBS 133181
Diaporthe schini LGMF910
Diaporthe masirevicii BRIP 57892a
Diaporthe masirevicii BRIP 57330
Diaporthe helianthi CBS 592.81
Diaporthe helianthi CBS 344.94
Diaporthe phaseolorum BDKHADRA-2
Diaporthe phaseolorum CBS 139281
Diaporthe phaseolorum CBS 113425
DF2
DF12
Diaporthe ueckerae FAU656
Diaporthe ueckerae FAU659
Diaporthe ueckerae FAU658
Diaporthe miriciae BRIP 55662c
Diaporthe miriciae BRIP 56918a
Diaporthe miriciae BRIP 54736j
DF7
DFP2
Diaporthe tectonendophytica MFLUCC 13-0471
Diaporthe unshiuensis ZJUD51
Diaporthe unshiuensis ZJUD50
Diaporthe unshiuensis ZJUD52
Diaporthe sojae FAU599
Diaporthe sojae FAU644
Paraphoma chlamydocopiosa BRIP 65168
Diaporthella corylina CBS 121124
Lasiodiplodia pseudotheobromae CBS116459
Nigrospora musae CBS 319.34
Arthrinium obovatum LC4940
0.05

Supplementary material Maximum-likelihood tree of *Diaporthe* species isolated from stem grey blight of *H. polyrhizus* based on ITS region with 1000 bootstrap replications. Isolates of the present study are presented in bold and other fungal genera are used as an outgroup. Bootstrap values are shown at the node and the scale bar indicates the number of substitutions per position.

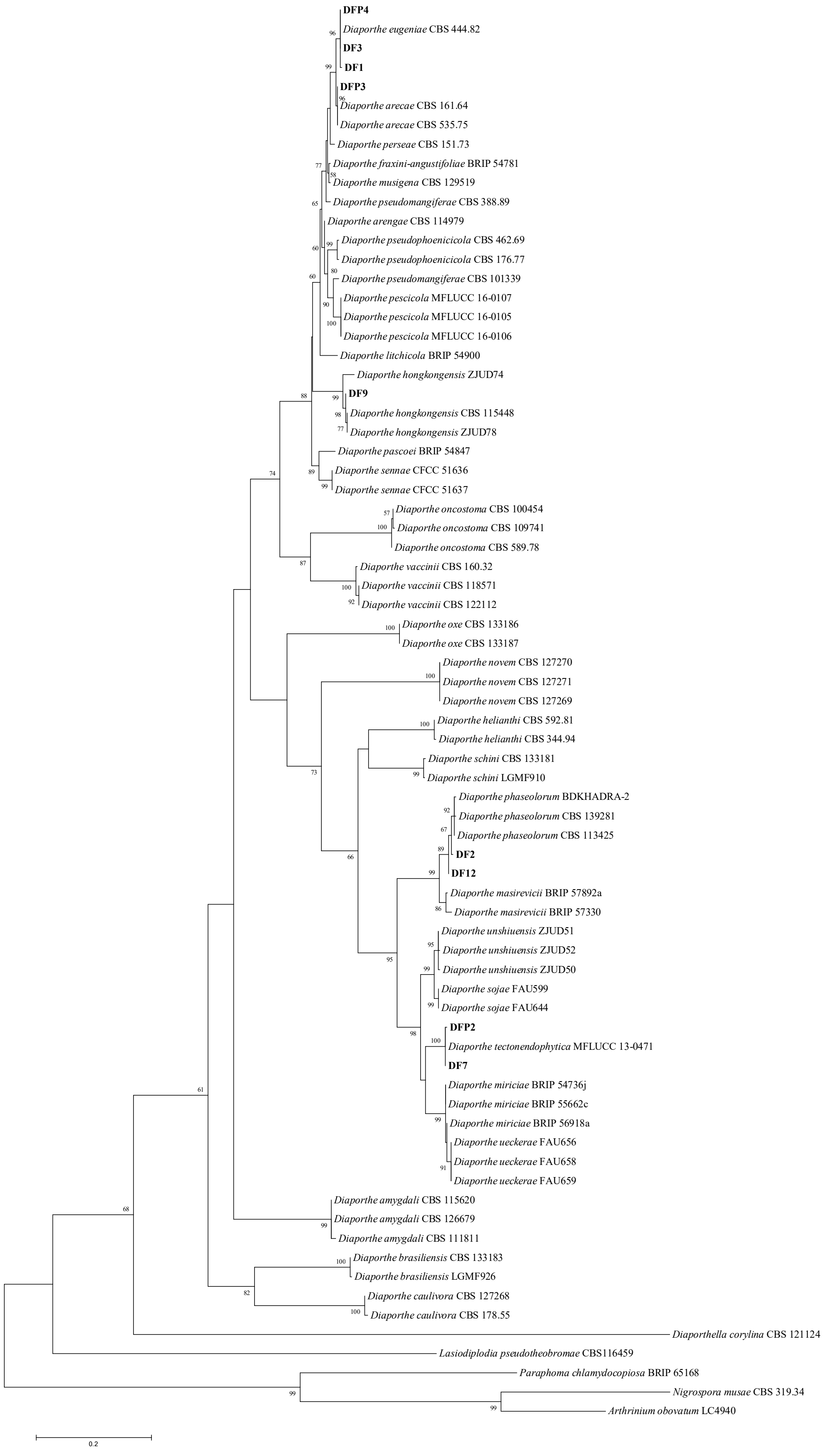
DFP4
Diaporthe eugeniae CBS 444.82
DF3
DF1
DFP3
Diaporthe arecae CBS 161.64
Diaporthe arecae CBS 535.75
Diaporthe perseae CBS 151.73
Diaporthe fraxini-angustifoliae BRIP 54781
Diaporthe musigena CBS 129519
Diaporthe pseudomangiferae CBS 388.89
Diaporthe arengae CBS 114979
Diaporthe pseudophoenicicola CBS 462.69
Diaporthe pseudophoenicicola CBS 176.77
Diaporthe pseudomangiferae CBS 101339
Diaporthe pescicola MFLUCC 16-0107
Diaporthe pescicola MFLUCC 16-0105
Diaporthe pescicola MFLUCC 16-0106
Diaporthe litchicola BRIP 54900
Diaporthe hongkongensis ZJUD74
DF9
Diaporthe hongkongensis CBS 115448
Diaporthe hongkongensis ZJUD78
Diaporthe pascoei BRIP 54847
Diaporthe sennae CFCC 51636
Diaporthe sennae CFCC 51637
Diaporthe oncostoma CBS 100454
Diaporthe oncostoma CBS 109741
Diaporthe oncostoma CBS 589.78
Diaporthe vaccinii CBS 160.32
Diaporthe vaccinii CBS 118571
Diaporthe vaccinii CBS 122112
Diaporthe oxe CBS 133186
Diaporthe oxe CBS 133187
Diaporthe novem CBS 127270
Diaporthe novem CBS 127271
Diaporthe novem CBS 127269
Diaporthe helianthi CBS 592.81
Diaporthe helianthi CBS 344.94
Diaporthe schini CBS 133181
Diaporthe schini LGMF910
Diaporthe phaseolorum BDKHADRA-2
Diaporthe phaseolorum CBS 139281
Diaporthe phaseolorum CBS 113425
DF2
DF12
Diaporthe masirevicii BRIP 57892a
Diaporthe masirevicii BRIP 57330
Diaporthe unshiuensis ZJUD51
Diaporthe unshiuensis ZJUD52
Diaporthe unshiuensis ZJUD50
Diaporthe sojae FAU599
Diaporthe sojae FAU644
DFP2
Diaporthe tectonendophytica MFLUCC 13-0471
DF7
Diaporthe miriciae BRIP 54736j
Diaporthe miriciae BRIP 55662c
Diaporthe miriciae BRIP 56918a
Diaporthe ueckerae FAU656
Diaporthe ueckerae FAU658
Diaporthe ueckerae FAU659
Diaporthe amygdali CBS 115620
Diaporthe amygdali CBS 126679
Diaporthe amygdali CBS 111811
Diaporthe brasiliensis CBS 133183
Diaporthe brasiliensis LGMF926
Diaporthe caulivora CBS 127268
Diaporthe caulivora CBS 178.55
Diaporthella corylina CBS 121124
Lasiodiplodia pseudotheobromae CBS116459
Paraphoma chlamydocopiosa BRIP 65168
Nigrospora musae CBS 319.34
Arthrinium obovatum LC4940
0.2

Supplementary material Maximum-likelihood tree of *Diaporthe* species isolated from stem grey blight of *H. polyrhizus* based on TEF1-α gene with 1000 bootstrap replications. Isolates of the present study are presented in bold and other fungal genera are used as an outgroup. Bootstrap values are shown at the node and the scale bar indicates the number of substitutions per position.

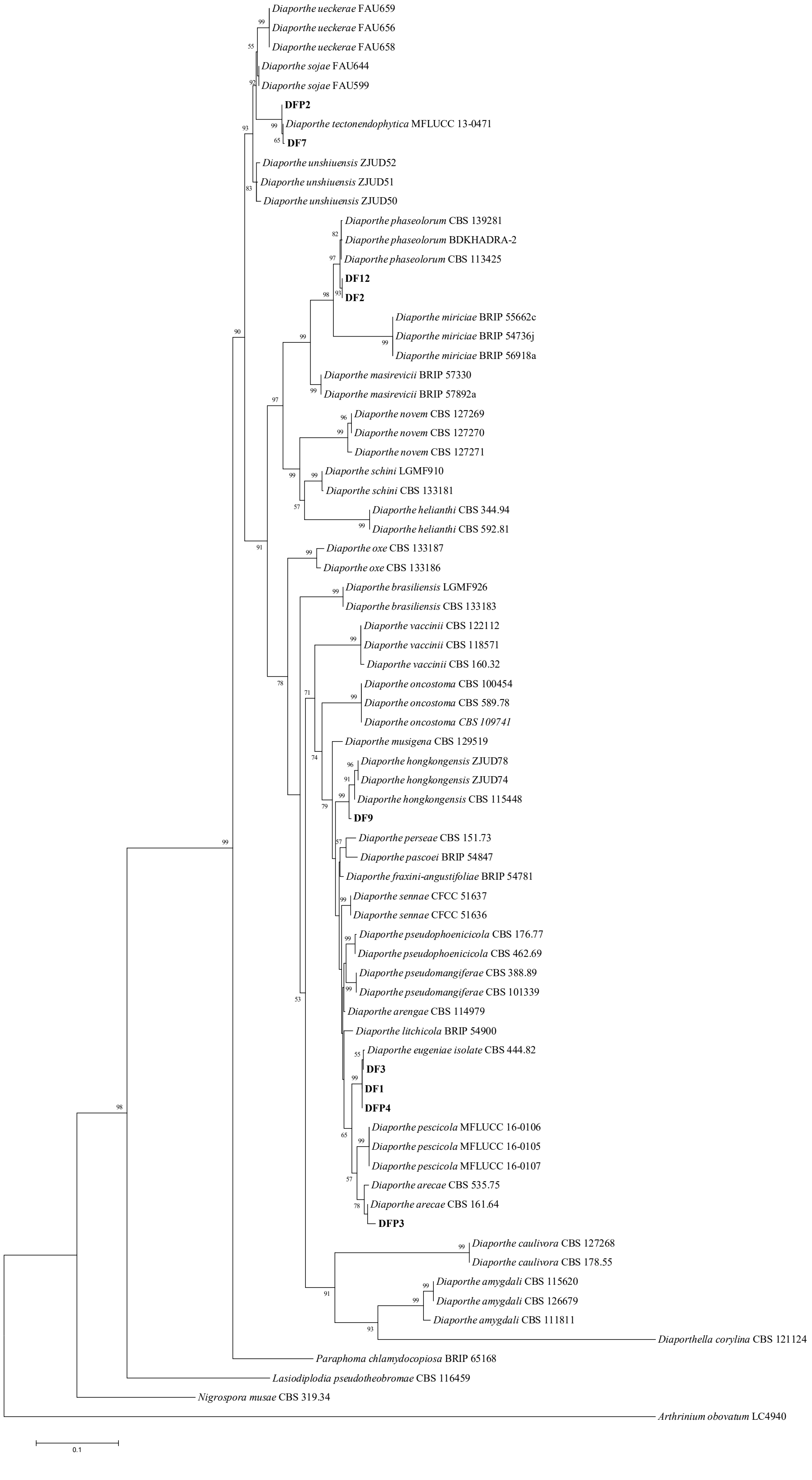
Diaporthe ueckerae FAU659
Diaporthe ueckerae FAU656
Diaporthe ueckerae FAU658
Diaporthe sojae FAU644
Diaporthe sojae FAU599
DFP2
Diaporthe tectonendophytica MFLUCC 13-0471
DF7
Diaporthe unshiuensis ZJUD52
Diaporthe unshiuensis ZJUD51
Diaporthe unshiuensis ZJUD50
Diaporthe phaseolorum CBS 139281
Diaporthe phaseolorum BDKHADRA-2
Diaporthe phaseolorum CBS 113425
DF12
DF2
Diaporthe miriciae BRIP 55662c
Diaporthe miriciae BRIP 54736j
Diaporthe miriciae BRIP 56918a
Diaporthe masirevicii BRIP 57330
Diaporthe masirevicii BRIP 57892a
Diaporthe novem CBS 127269
Diaporthe novem CBS 127270
Diaporthe novem CBS 127271
Diaporthe schini LGMF910
Diaporthe schini CBS 133181
Diaporthe helianthi CBS 344.94
Diaporthe helianthi CBS 592.81
Diaporthe oxe CBS 133187
Diaporthe oxe CBS 133186
Diaporthe brasiliensis LGMF926
Diaporthe brasiliensis CBS 133183
Diaporthe vaccinii CBS 122112
Diaporthe vaccinii CBS 118571
Diaporthe vaccinii CBS 160.32
Diaporthe oncostoma CBS 100454
Diaporthe oncostoma CBS 589.78
Diaporthe oncostoma CBS 109741
Diaporthe musigena CBS 129519
Diaporthe hongkongensis ZJUD78
Diaporthe hongkongensis ZJUD74
Diaporthe hongkongensis CBS 115448
DF9
Diaporthe perseae CBS 151.73
Diaporthe pascoei BRIP 54847
Diaporthe fraxini-angustifoliae BRIP 54781
Diaporthe sennae CFCC 51637
Diaporthe sennae CFCC 51636
Diaporthe pseudophoenicicola CBS 176.77
Diaporthe pseudophoenicicola CBS 462.69
Diaporthe pseudomangiferae CBS 388.89
Diaporthe pseudomangiferae CBS 101339
Diaporthe arengae CBS 114979
Diaporthe litchicola BRIP 54900
Diaporthe eugeniae isolate CBS 444.82
DF3
DF1
DFP4
Diaporthe pescicola MFLUCC 16-0106
Diaporthe pescicola MFLUCC 16-0105
Diaporthe pescicola MFLUCC 16-0107
Diaporthe arecae CBS 535.75
Diaporthe arecae CBS 161.64
DFP3
Diaporthe caulivora CBS 127268
Diaporthe caulivora CBS 178.55
Diaporthe amygdali CBS 115620
Diaporthe amygdali CBS 126679
Diaporthe amygdali CBS 111811
Diaporthella corylina CBS 121124
Paraphoma chlamydocopiosa BRIP 65168
Lasiodiplodia pseudotheobromae CBS 116459
Nigrospora musae CBS 319.34
Arthrinium obovatum LC4940
0.1

Supplementary material Maximum-likelihood tree of *Diaporthe* species isolated from stem grey blight of *H. polyrhizus* based on β-tubulin gene with 1000 bootstrap replications. Isolates of the present study are presented in bold and other fungal genera are used as an outgroup. Bootstrap values are shown at the node and the scale bar indicates the number of substitutions per position.
